# Evaluating the relationship between ciprofloxacin prescription and non-susceptibility in *Salmonella* Typhi in Blantyre, Malawi: an observational study

**DOI:** 10.1016/S2666-5247(23)00327-0

**Published:** 2024-03

**Authors:** Philip M Ashton, Angeziwa Chunga Chirambo, James E Meiring, Priyanka D Patel, Maurice Mbewe, Niza Silungwe, Kenneth Chizani, Happy Banda, Robert S Heyderman, Zoe A Dyson, Peter MacPherson, Marc Y R Henrion, STRATAA Study Group, Prasanta Kumar Biswas, Md Amiruli Islam Bhuiyan, Christoph Blohmke, Thomas C Darton, Christiane Dolecek, Sabina Dongol, Yama Farooq, Jennifer Hill, Nhu Tran Hoang, Tikhala Makhaza Jere, Harrison Msuku, Tran Vu Thieu Nga, Rose Nkhata, Sadia Isfat Ara Rahman, Nazia Rahman, Neil J Saad, Trinh Van Tan, Deus Thindwa, Merryn Voysey, Richard Wachepa, Andrew Pollard, Kathryn E Holt, Melita A Gordon

**Affiliations:** k Malawi-Liverpool Wellcome Programme, Blantyre, Malawi; l International Centre for Diarrhoeal Disease Research, Dhaka, Bangladesh; m Oxford Vaccine Group, Department of Paediatrics, University of Oxford, and the NIHR Oxford Biomedical Research Centre, Oxford, UK; n Nuffield Department of Medicine, Centre for Tropical Medicine and Global Health, University of Oxford, Oxford, UK; o Oxford University Clinical Research Unit, Patan Academy of Health Sciences, Kathmandu, Nepal; p The Hospital for Tropical Diseases, Wellcome Trust Major Overseas Programme, Oxford University Clinical Research Unit, Ho Chi Minh City, Viet Nam; q Department of Epidemiology of Microbial Diseases and the Public Health Modeling Unit, Yale School of Public Health, Yale University, New Haven, CT, USA; a Malawi-Liverpool-Wellcome Programme, Blantyre, Malawi; b Institute of Infection, Veterinary & Ecological Sciences, University of Liverpool, Liverpool, UK; c Department of Medical Laboratory Sciences, Kamuzu University of Health Sciences, Blantyre, Malawi; d Department of Infection, Immunity and Cardiovascular Disease, University of Sheffield, Sheffield, UK; e Research Department of Infection, Division of Infection and Immunity, University College London, London, UK; f Faculty of Infectious and Tropical Diseases, London School of Hygiene & Tropical Medicine, London, UK; g Department of Infectious Diseases, Central Clinical School, Monash University, Melbourne, VIC, Australia; h Wellcome Sanger Institute, Wellcome Genome Campus, Hinxton, UK; i School of Health & Wellbeing, University of Glasgow, Glasgow, UK; j Department of Clinical Sciences, Liverpool School of Tropical Medicine, Liverpool, UK

## Abstract

**Background:**

Ciprofloxacin is the first-line drug for treating typhoid fever in many countries in Africa with a high disease burden, but the emergence of non-susceptibility poses a challenge to public health programmes. Through enhanced surveillance as part of vaccine evaluation, we investigated the occurrence and potential determinants of ciprofloxacin non-susceptibility in Blantyre, Malawi.

**Methods:**

We conducted systematic surveillance of typhoid fever cases and antibiotic prescription in two health centres in Blantyre, Malawi, between Oct 1, 2016, and Oct 31, 2019, as part of the STRATAA and TyVAC studies. In addition, blood cultures were taken from eligible patients presenting at Queen Elizabeth Central Hospital, Blantyre, as part of routine diagnosis. Inclusion criteria were measured or reported fever, or clinical suspicion of sepsis. Microbiologically, we identified *Salmonella enterica* serotype Typhi (*S* Typhi) isolates with a ciprofloxacin non-susceptible phenotype from blood cultures, and used whole-genome sequencing to identify drug-resistance mutations and phylogenetic relationships. We constructed generalised linear regression models to investigate associations between the number of ciprofloxacin prescriptions given per month to study participants and the proportion of *S* Typhi isolates with quinolone resistance-determining region (QRDR) mutations in the following month.

**Findings:**

From 46 989 blood cultures from Queen Elizabeth Central Hospital, 502 *S* Typhi isolates were obtained, 30 (6%) of which had either decreased ciprofloxacin susceptibility, or ciprofloxacin resistance. From 11 295 blood cultures from STRATAA and TyVAC studies, 241 microbiologically confirmed cases of typhoid fever were identified, and 198 isolates from 195 participants sequenced (mean age 12·8 years [SD 10·2], 53% female, 47% male). Between Oct 1, 2016, and Aug 31, 2019, of 177 typhoid fever cases confirmed by whole-genome sequencing, four (2%) were caused by *S* Typhi with QRDR mutations, compared with six (33%) of 18 cases between Sept 1 and Oct 31, 2019. This increase was associated with a preceding spike in ciprofloxacin prescriptions. Every additional prescription of ciprofloxacin given to study participants in the preceding month was associated with a 4·2% increase (95% CI 1·8–7·0) in the relative risk of isolating *S* Typhi with a QRDR mutation (p=0·0008). Phylogenetic analysis showed that *S* Typhi isolates with QRDR mutations from September and October, 2019, belonged to two distinct subclades encoding two different QRDR mutations, and were closely related (4–10 single-nucleotide polymorphisms) to susceptible *S* Typhi endemic to Blantyre.

**Interpretation:**

We postulate a causal relationship between increased ciprofloxacin prescriptions and an increase in fluoroquinolone non-susceptibility in *S* Typhi. Decreasing ciprofloxacin use by improving typhoid diagnostics, and reducing typhoid fever cases through the use of an efficacious vaccine, could help to limit the emergence of resistance.

**Funding:**

Wellcome Trust, Bill & Melinda Gates Foundation, and National Institute for Health and Care Research (UK).

## Introduction

*Salmonella enterica* serotype Typhi (*S* Typhi) is the causative agent of typhoid fever, a systemic infectious disease. Globally, in 2017, there were an estimated 10·9 million cases of typhoid fever (95% CI 9·3–12·6), resulting in 116 800 deaths (65 400–187 700), mainly in countries in south and southeast Asia and sub-Saharan Africa.^1^

**Research in context**

**Evidence before this study**

Current knowledge of the drivers of antimicrobial resistance in settings with a high burden of infectious diseases is largely inferred from data from countries with a low burden of infectious diseases. We searched PubMed on April 6, 2023, for articles reporting on the association between fluoroquinolone use and the emergence of fluoroquinolone resistance in countries with a high burden of infectious diseases (see appendix 1 p 1 for full query). Our search terms included *Escherichia coli, Salmonella,* Enterobacteriaceae, *Klebsiella,* and enteric bacteria, and we excluded only tuberculosis. We identified three studies from African countries that identified an association between ciprofloxacin use and ciprofloxacin non-susceptibility in *E coli*. None of these three papers investigated ciprofloxacin resistance in isolates causing invasive disease.

**Added value of this study**

We identified an association between the proportion of *Salmonella enterica* serotype Typhi (*S* Typhi) with a non-synonymous mutation in the quinolone resistance-determining region (QRDR) and the number of ciprofloxacin prescriptions in the previous month. We used genome sequencing to identify that the *S* Typhi isolates with the QRDR mutations evolved locally from the endemic *S* Typhi population in Blantyre, Malawi, and were not the result of importations from other countries.

**Implications of all the available evidence**

Although the direction of causality is difficult to establish in an ecological study design such as ours, the implication of the available evidence is that increases in ciprofloxacin use can rapidly cause the emergence of resistance in *S* Typhi. As ciprofloxacin is still the first-line therapy for multidrug-resistant typhoid fever in many African countries, this information is highly pertinent for health policy makers. Improved typhoid diagnostics and the introduction of efficacious vaccines, such as typhoid conjugate vaccines, could mitigate this adverse outcome.

Drug resistance is a major problem in *S* Typhi infection, leading to worse outcomes for patients and having substantial implications for overstretched health systems in low-income and middle-income countries. At least 75% of isolates of *S* Typhi in Africa have been identified to be multidrug-resistant (MDR), with resistance to first-line antibiotics including ampicillin, chloramphenicol, and co-trimoxazole.^2^ The MDR phenotype was associated with the emergence of the H58 lineage in south and southeast Asia and east and southern Africa.^3^^,^^4^

*Salmonellae* that are resistant to fluoroquinolones (eg, ciprofloxacin) are on WHO’s list of global priority antibiotic-resistant pathogens. In Asia, *S* Typhi with decreased ciprofloxacin susceptibility (DCS; defined as a ciprofloxacin minimum inhibitory concentration [MIC] ≥0·12 μg/mL to <1 μg/mL) accounted for more than 60% of typhoid fever cases in Asia in 2011–15, which led to the adoption of azithromycin and third-generation cephalosporins as the first-line therapy for typhoid fever in this region.^2^ In Africa, the proportion of infections with *S* Typhi with DCS is below 20%,^2^ although there is substantial variation between countries.^5^, ^6^, ^7^, ^8^, ^9^, ^10^ In Zimbabwe, the proportion of *S* Typhi isolates referred to the national reference laboratory that were resistant to ciprofloxacin (with ciprofloxacin MICs ≥1 μg/mL) increased from 4% to 22% between 2012 and 2017,^11^ while a large outbreak of typhoid fever in the city of Harare showed ciprofloxacin resistance in *S* Typhi isolates from 33% of hospitalised patients.^12^ Whole-genome sequencing showed that the ciprofloxacin non-susceptibility, or resistance, was due to plasmid-mediated quinolone-resistance genes, in some cases combined with quinolone resistance-determining region (QRDR) mutations.^13^ In Malawi, an enhanced passive surveillance study in the Ndirande urban township area of the city of Blantyre found that 92% of *S* Typhi isolates were MDR, but only 0·9% of isolates had DCS.^14^ These findings are consistent with a previous report from a hospital cohort in which none of 176 *S* Typhi isolates had DCS.^4^

Against a background of a low rate of ciprofloxacin non-susceptibility in *S* Typhi in Malawi, we investigated the genomic and epidemiological features of a cluster of *S* Typhi with QRDR mutations isolated from Blantyre, Malawi, in September to October, 2019.

## Methods

### Study design and participants

Participants were enrolled either from the community-based secondary health centres in the Ndirande or Zingwangwa townships (between Oct 1, 2016, and Oct 31, 2019) or from Queen Elizabeth Central Hospital (between Jan 1, 2019, and Jan 31, 2022), in Blantyre, Malawi.^15^^,^^16^ Further information about the setting can be found in appendix 1 (pp 1–2).

Participants who contributed bacterial isolates to this study were recruited from two main sources. The first source of participants was the routine blood culture service provided to Queen Elizabeth Central Hospital (a tertiary-level hospital) by the Malawi-Liverpool-Wellcome Programme. This service is for adults (aged ≥16 years) admitted to the hospital with axillary temperature over 37·5°C or with clinical suspicion of sepsis, and for children (aged <16 years) who are malaria slide negative, or malaria slide positive and critically ill, or with clinical suspicion of sepsis. We included all *S* Typhi isolates obtained by this service from Jan 1, 2019, to Jan 31, 2022, for which pefloxacin disk diffusion susceptibility testing results were available. From Nov 1, 2019, all pefloxacin screening was done routinely by the Malawi-Liverpool-Wellcome Programme blood culture service; isolates from before this date were recovered from the programme’s –80°C freezer archive and tested as part of the current study.

The second source of participants was the Strategic Typhoid Alliance Across Africa & Asia (STRATAA) and Typhoid Vaccine Acceleration Consortium (TyVAC) studies, the detailed methods of which have been previously published.^14^, ^15^, ^16^ The primary goal of STRATAA was to quantify the incidence of typhoid fever at three sites in Asia and Africa, which was done by passive surveillance at selected secondary-care outpatient health centres at those sites. The primary goal of TyVAC was to assess the safety and efficacy of the typhoid conjugate vaccine. Secondary-care outpatient health centre-based passive surveillance was used to detect cases of typhoid fever among TyVAC participants. Cases were defined by microbiologically confirmed *S* Typhi bloodstream infection. Relevant details are presented in appendix 1 (pp 2–3). Ciprofloxacin prescriptions given to study participants by either government or study staff were recorded in the clinical research form. Fluoroquinolones are considered to be the most appropriate first-line treatment for adults and children who are able to take oral medication for typhoid,^17^ and are available at both secondary-level and tertiary-level health facilities for this purpose in accordance with Malawi national guidelines.^18^

This research was approved by the appropriate research ethics committees in Malawi (College of Medicine Research Ethics Committee and National Health Science Research Committee) and at the University of Liverpool (Liverpool, UK). Participants or their guardians provided written informed consent.

### Procedures

The study team had access to all isolates reported here and, where the procedures were not done immediately upon isolation, isolates were stored on beads at –80°C until the time of the procedure. The pefloxacin disk diffusion method was used to test all isolates in this study to identify those with altered susceptibility to fluoroquinolones according to the European Committee on Antimicrobial Susceptibility Testing guidelines.^19^ For any isolates with reduced susceptibility to pefloxacin, the ciprofloxacin MIC was confirmed by E-test (appendix 1 p 3).

For the sequencing of isolates from the STRATAA and TyVAC studies, isolates were cultured overnight on XLD agar at 37°C and genomic DNA was extracted with the Wizard Genomic DNA Extraction Kit (Promega, Madison, WI, USA) following the manufacturer’s recommendations. Genomic DNA was shipped to the Wellcome Sanger Institute for indexed whole-genome sequencing on an Illumina HiSeq 2500 platform (Illumina, San Diego, CA, USA) to generate paired-end reads of 100 bp in length. A subset of genome sequences from participants enrolled in STRATAA between Oct 1, 2016, and Aug 31, 2019, have been reported in the multisite analysis for that study.^20^ Bioinformatics methods were used to identify the subclades of *S* Typhi, identify antimicrobial resistance (AMR) genes and mutations, measure single-nucleotide polymorphism (SNP) distances, and generate maximum-likelihood phylogenies (appendix 1 p 4).

### Statistical analysis

To investigate the association between the proportion of total sequencing-confirmed typhoid fever cases with QRDR mutations and the total number of ciprofloxacin prescriptions in the preceding month, we constructed a generalised linear regression model with a binomial distribution. No covariables were used as there were no variables available to our study that changed over the study period and that could have had a notable influence on the proportion of *S* Typhi isolates with QRDR mutations detected, other than the number of doses of ciprofloxacin prescribed. By using proportions, the binomial model accounts for the single largest contributor to more QRDR cases, the number of isolates, and the difference in recruitment criteria between centres. The data were complete with respect to the variables included in the analysis (the proportion of *S* Typhi with QRDR mutations and monthly ciprofloxacin prescriptions were available for each month), and thus no specific methods were needed to account for missing data. The binomial 95% CIs for percentage proportions were calculated using the prop.test function in R. We have interpreted odds ratios (ORs) as relative risk given the rarity of the outcome (*S* Typhi with QRDR mutations).

All analyses were done with R (version 4.1.0); further details are available in appendix 1 (p 5).

### Role of the funding source

The funders of the study had no role in study design, data collection, data analysis, data interpretation, or writing of the report.

## Results

**Figure 1:**
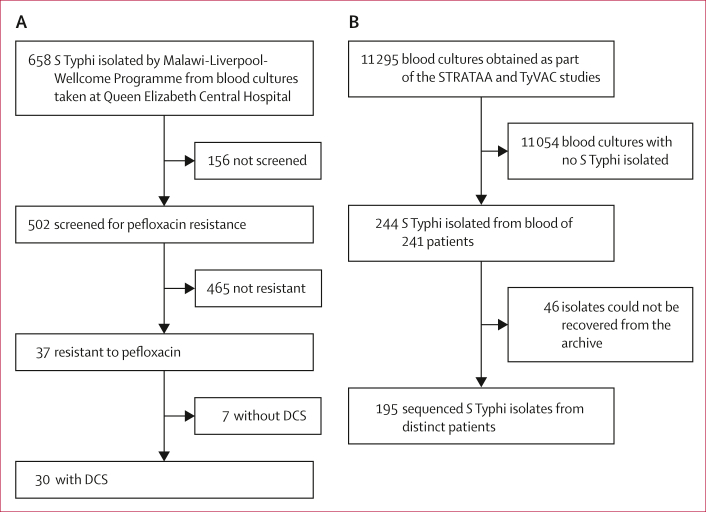
Flow diagram of samples included in study (A) *S* Typhi isolates from Queen Elizabeth Central Hospital that had only antimicrobial resistance phenotyping done. (B) *S* Typhi isolates from the STRATAA and TyVAC studies. DCS=decreased ciprofloxacin susceptibility. *S* Typhi=*Salmonella enterica* serotype Typhi.

**Figure 2:**
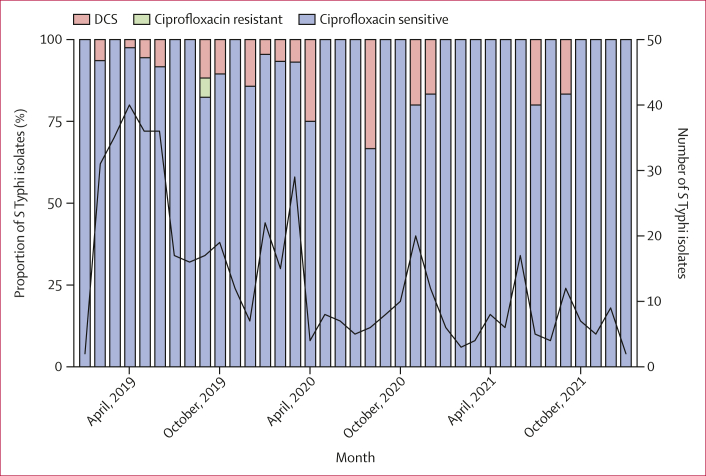
Ciprofloxacin-sensitive, DCS, or ciprofloxacin-resistant phenotypes among *S* Typhi isolated from Queen Elizabeth Central Hospital, by month Ciprofloxacin susceptibility phenotypes were determined by minimum inhibitory concentrations: sensitive ≤0·06 μg/mL; DCS ≥0·12 μg/mL to <1 μg/mL; and resistant ≥1 μg/mL. The total number of *S* Typhi isolated from Queen Elizabeth Central Hospital is plotted as a black line. DCS=decreased ciprofloxacin susceptibility. *S* Typhi=*Salmonella enterica* serotype Typhi.

From 46 989 patients presenting at Queen Elizabeth Central Hospital between Jan 1, 2019 and Jan 31, 2022, we screened 502 *S* Typhi isolates for resistance to pefloxacin, and identified 37 isolates (7%) that were resistant (figure 1A). 30 isolates (6% [95% CI 4–9] of total) were confirmed to have either DCS (n=29) or ciprofloxacin resistance (n=1; figure 2).

**Figure 3:**
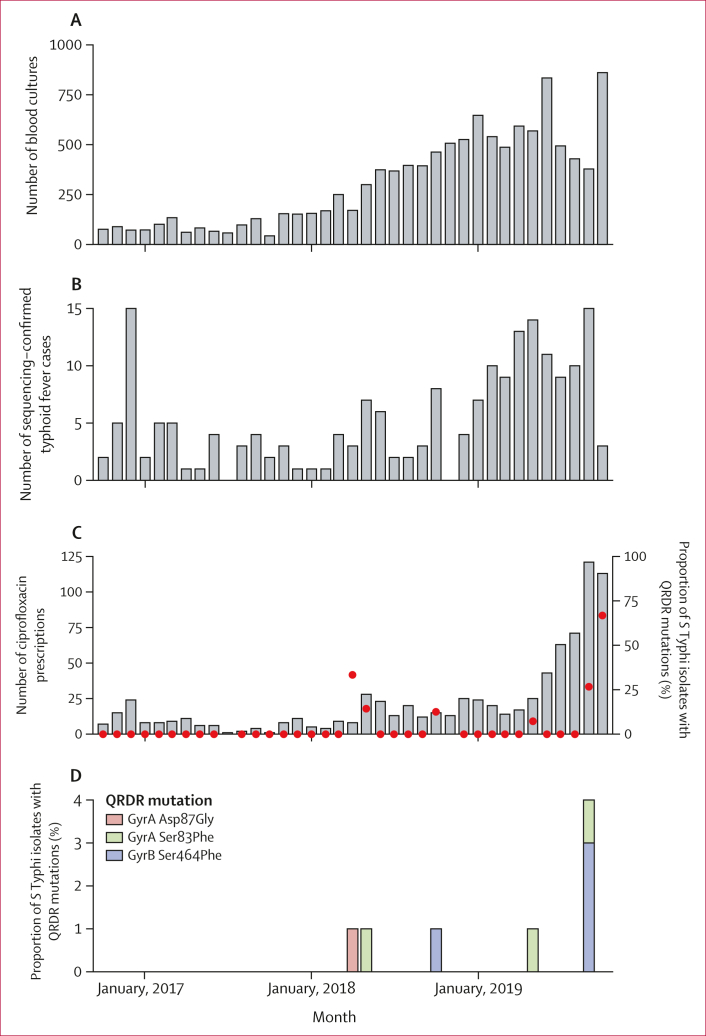
Results of enhanced passive surveillance at the Ndirande and Zingwangwa health centres (A) Total number of blood cultures taken from eligible participants. (B) Total sequencing-confirmed typhoid fever cases. (C) Number of ciprofloxacin prescriptions (grey bars) and the proportion of *S* Typhi isolates with sequencing-confirmed QRDR mutations (red dots). (D) Frequency of *S* Typhi with each QRDR mutation. *S* Typhi=*Salmonella enterica* serotype Typhi. QRDR=quinolone resistance-determining region.

Between Oct 1, 2016, and Oct 31, 2019, we conducted enhanced passive surveillance for *S* Typhi at the Ndirande and Zingwangwa health centres. From 11 295 blood cultures, we identified 244 *S* Typhi isolates from 241 patients (figure 1B). Primary results from the sequencing of isolates obtained as part of the STRATAA study between Oct 1, 2016, and Aug 31, 2019, are reported elsewhere.^20^ Here, we report a combined analysis of the 141 *S* Typhi genomes from Malawi previously published by Dyson and colleagues,^20^ and 57 novel genomes from 54 patients, extending coverage of genomic surveillance to Oct 31, 2019. In total, from patients recruited between Oct 1, 2016, and Oct 31, 2019, we sequenced 198 of 244 *S* Typhi isolates from 195 of 241 patients (figure 1; appendix 1 pp 8–9) and identified *S* Typhi from ten patients with non-synonymous mutations in the QRDR (figure 3). The mean age of patients from whom isolates were sequenced was 12·8 years (SD 10·2), 53% of patients were female, and 47% were male. All isolates that could be recovered from the Malawi-Liverpool-Wellcome Programme culture archive were sequenced. No plasmid-mediated quinolone-resistance mechanisms were identified in *S* Typhi isolates from Blantyre.

In the first half of 2019, there was an increase in cases of MDR typhoid fever. Between October, 2016, and December, 2018, a median of 3 sequencing-confirmed cases (IQR 1·5–4·5) of typhoid fever were identified per month, increasing to 10 cases (9–12·5) per month between January and October, 2019 (figure 3B). Following this increase in MDR typhoid fever, the number of ciprofloxacin prescriptions increased in the second half of 2019, from a median of 11 (range 1–28; IQR 6·75–17·75) monthly prescriptions between October, 2016, and May, 2019, to 71 (range 43–121; IQR 63–113) monthly prescriptions between June and October, 2019 (figure 3C). Not long after the increase in ciprofloxacin prescriptions, we noted an increase in DCS *S* Typhi: between October, 2016, and August, 2019, four (2% [95% CI 0·006–6]) of 177 cases of typhoid fever were caused by *S* Typhi with QRDR mutations, whereas from September to October, 2019, six (33% [14–58]) of 18 cases of typhoid fever were caused by *S* Typhi with QRDR mutations (Fisher’s exact test p<0·0001; figure 3C). No other antibiotic showed a trend in prescription similar to that of ciprofloxacin (appendix 1 p 10).

Between October, 2016, and August, 2019, three different QRDR mutations were identified: GyrA Ser83Phe (n=2), GyrA Asp87Gly (n=1), and GyrB Ser464Phe (n=1). During September and October, 2019, two different mutations were identified: GyrB Ser464Phe (n=5) and GyrA Ser83Phe (n=1; figure 3D). Nine of the ten samples with QRDR mutations had a DCS phenotype.

Regression modelling showed that each additional prescription of ciprofloxacin given to STRATAA and TyVAC participants was associated with a 4·2% increase (95% CI 1·8–7·0; p=0·0008) in the relative risk of isolating *S* Typhi with a QRDR mutation in the subsequent month. The number of blood cultures taken was not significantly associated with the number of *S* Typhi with QRDR mutations isolated in that month (OR 1·00 [95% CI 1·00–1·00], p=0·22). As there were two centres enrolling participants in our study, we did a sensitivity analysis to assess the association between ciprofloxacin prescriptions and the proportion of *S* Typhi with QRDR mutations at each centre separately. As only one case with *S* Typhi with QRDR mutation was identified at the Zingwangwa health centre during the study period, no statistically meaningful analysis could be done. However, importantly, there were no sustained preceding changes in ciprofloxacin prescriptions at this site (appendix 1 p 11). Six cases with *S* Typhi with QRDR mutations were isolated from Ndirande from September to October, 2019. The proportion of *S* Typhi with QRDR mutations was significantly associated with the number of ciprofloxacin prescriptions in the preceding month (4·8% increased relative risk [95% CI 2·5–7·6], p=0·0008; appendix 1 p 12). There was no association between the number of ciprofloxacin prescriptions and the proportion of *S* Typhi isolates with DCS or ciprofloxacin resistance as detected by E-test at Queen Elizabeth Central Hospital (OR 1·01 [95% CI 0·99–1·02], p=0·32).

We did a phylogenetic analysis of the 195 *S* Typhi isolates from different patients reported here, combined with 112 *S* Typhi isolates from Blantyre from previously published work (appendix 1 p 12).^4^ Five of the six *S* Typhi isolates from Ndirande in September and October, 2019 formed a subclade of near-identical isolates (median SNP pairwise distance 0 SNPs [IQR 0–0], 10 SNPs to nearest neighbour) that shared the GyrB Ser464Phe amino acid substitution. The sixth isolate was unrelated to the other five and encoded a GyrA Ser83Phe amino acid substitution; its closest phylogenetic neighbour (four SNPs) was isolated in May, 2019, from the Ndirande health centre and also encoded a GyrA Ser83Phe amino acid substitution.

**Figure 4:**
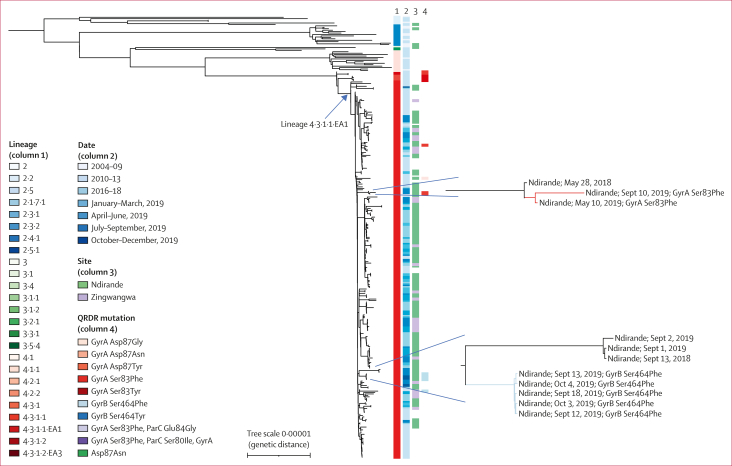
Phylogenetic tree of 307 *S* Typhi from Blantyre (one genome per patient) Tree branches in insets are coloured according to the QRDR mutation observed descending from that branch (corresponding to column 4). *S* Typhi=*Salmonella enterica* serotype Typhi. QRDR=quinolone resistance-determining region.

**Figure 5:**
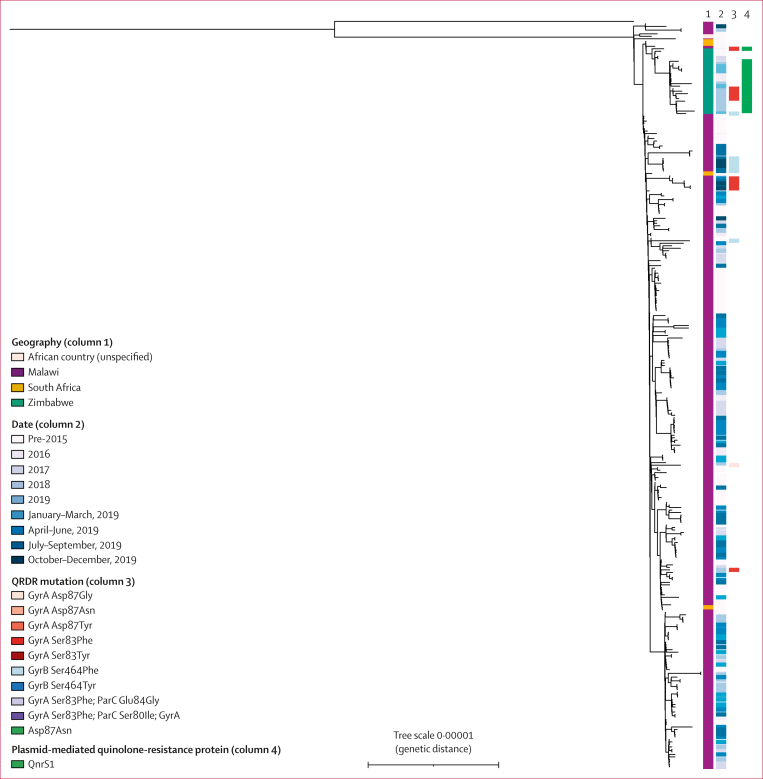
Phylogenetic tree of 299 *S* Typhi genomes from the Malawian and Zimbabwean subclade and an outlier *S* Typhi=*Salmonella enterica* serotype Typhi. QRDR=quinolone resistance-determining region.

To investigate the hypothesis that the *S* Typhi with QRDR mutations were imported from outside Blantyre, we derived a maximum-likelihood phylogenetic tree of 307 genomes from Malawi alongside an additional 1970 *S* Typhi genomes from outside of Malawi from published studies. As the subclades of interest all belonged to H58, we present a subtree of 1176 H58 genomes (figure 4).^3^^,^^5^^,^^12^ 261 (85%) of 307 genomes from Malawi formed a paraphyletic clade belonging to L4.3.1.1.EA1, which also included 27 genomes from Zimbabwe, four genomes from South Africa, and one genome from an unspecified African country. The Zimbabwean genomes included isolates encoding both the QnrS1 plasmid-mediated quinolone-resistance protein and QRDR mutations. An additional maximum-likelihood phylogeny was constructed, focusing on this subclade (figure 5), which confirmed that the 27 genomes from Zimbabwe formed a monophyletic clade and suggested no transmission of *S* Typhi with QRDR mutations between Zimbabwe and Malawi in this dataset. The most closely related genomes to the Blantyre *S* Typhi with QRDR mutations were QRDR mutation-negative *S* Typhi from Blantyre.

## Discussion

Within an established vaccine trial and epidemiological surveillance study in Blantyre, Malawi, a setting with a background of low ciprofloxacin resistance, we found that each additional prescription of ciprofloxacin given to participants was associated with a 4·2% increase in the relative risk of isolating *S* Typhi with a QRDR mutation in the subsequent month. Placing our genomes into a broader genomic context revealed that the genomes most closely related to our DCS *S* Typhi were ciprofloxacin-susceptible *S* Typhi from Blantyre, and hence not the result of an introduction from another country.

Long-term blood culture surveillance at the outpatient secondary-care level is unusual in our setting. However, our surveillance platform enabled us to detect community cases of typhoid fever that influence empirical outpatient prescribing and the earliest emergence of QRDR mutations. In Blantyre, clinical microbiology tests are not standard, and prescriptions are not systematically recorded. This mirrors many primary or secondary health-care systems in countries in sub-Saharan Africa, leading to limited literature on the association between antibiotic use and resistance in the region.^21^, ^22^, ^23^

Genomic analysis enhanced our study by reliably identifying *S* Typhi with QRDR mutations; by contextualising our *S* Typhi findings within global data, confirming the DCS *S* Typhi's local emergence rather than foreign import; and by revealing that the *S* Typhi with QRDR mutations from September and October, 2019, belonged to distinct subclades with different mutations.

Our ecological study design examines the correlation between antimicrobial use and the emergence of resistance at the population level, rather than at the individual level, limiting our ability to establish definitive causality between ciprofloxacin use and QRDR emergence. However, we can compare the plausibility of forward causality (increased ciprofloxacin prescriptions leading to more *S* Typhi with QRDR mutations) against reverse causality (rise in *S* Typhi with QRDR mutations leading to more prescriptions). Forward causality is biologically plausible, supported by the significant association between the previous month's ciprofloxacin prescriptions and the proportion of mutated *S* Typhi. Although reverse causality might arise from prescribing in response to local AMR trends, it is unlikely that an increase in DCS *S* Typhi would prompt more ciprofloxacin prescriptions. Thus, forward causality appears most probable, but potential biases, such as external ciprofloxacin use, might exist.

As a sensitivity analysis, we investigated the association between ciprofloxacin prescriptions and the proportion of *S* Typhi with QRDR mutations at each of the two health centres. As the QRDR-mutated *S* Typhi were primarily isolated from the Ndirande health centre, there was only a significant association at this site, while there was neither any increase in ciprofloxacin prescriptions nor the emergence of QRDR-mutated *S* Typhi at the Zingwangwa health centre (appendix 1 p 11), strengthening the evidence for the forward causal association.

Most antibiotic use in Africa is empirical and often not prescribed by health professionals.^24^^,^^25^ In Malawi, a higher-than-average 64% of antibiotic prescriptions are done by health facilities,^26^ but a significant proportion are still informally obtained. Our study included ciprofloxacin prescriptions only from the STRATAA and TyVAC projects, introducing potential bias from untracked external use. However, ciprofloxacin is not commonly used as a non-prescription antibiotic in Malawi, with only 2–3% of people reporting using oral ciprofloxacin “frequently”, in contrast to 68% and 36% of people reporting frequent use of co-trimoxazole or amoxicillin, respectively.^26^^,^^27^ We cannot accurately estimate non-study-related prescribed ciprofloxacin use in our recruitment areas or broader Blantyre, but the low rate of DCS *S* Typhi isolated from people presenting at Queen Elizabeth Central Hospital is consistent with low community use of ciprofloxacin.

An important limitation of our study is the small sample size of *S* Typhi isolates with QRDR mutations due to our focus on their earliest emergence, reducing our power to discern an association. Hospital surveillance has not shown a widespread rise in DCS *S* Typhi. This finding could be due to Malawi’s low ciprofloxacin use, making interpretations challenging. Additionally, hospital-based detections of *S* Typhi declined after SARS-CoV-2 emergence, probably from reduced hospital visits owing to fear of the virus.

Models used in this study were not adjusted for any covariables because our outcome measure was the proportion of *S* Typhi isolates with QRDR mutations, and our methodology for identifying such isolates was consistent across the study period. If we had used the number of *S* Typhi isolates with QRDR mutations, then we would also need to account for the number of blood cultures done and the subtly different sampling frames of the two studies.

Our modelling revealed a significant positive association between ciprofloxacin prescriptions and the proportion of *S* Typhi isolates with QRDR mutations in the following month, against a background of low rates of DCS *S* Typhi in Blantyre (figure 2). This short lag time is in line with what other studies have found for the relationship between ciprofloxacin prescriptions and fluoroquinolone resistance.^28^, ^29^, ^30^

Uncontrolled use of antibiotics has long been blamed for the emergence of resistance to fluoroquinolones and other antibiotics in *S* Typhi in settings with a high burden of infectious disease,^2^^,^^31^ but empirical demonstrations of this relationship have been lacking. However, the association between ciprofloxacin use and resistance in *Enterobacteriaceae* in high-income countries, in hospitals and communities, has been well documented.^28^^,^^32^, ^33^, ^34^, ^35^, ^36^, ^37^, ^38^ Studies that showed that fluoroquinolone stewardship interventions reduced either the incidence or the rate of increase of fluoroquinolone resistance suggest that this is a causal association in some circumstances.^30^^,^^34^ Our study contributes to this evidence base by showing an association between prescribed antibiotics and proportion of *S* Typhi isolates with QRDR mutations in a community setting in sub-Saharan Africa, where there are few existing studies.^21^

If the association we have described is causal, it has important policy implications. Only around 1% of febrile patients in Blantyre have typhoid fever.^14^^,^^39^ Consequently, most ciprofloxacin courses in our study were not for typhoid-caused fevers. Two key niches were likely to increase *S* Typhi exposure to ciprofloxacin. The first such niche is chronic or asymptomatic carriers treated for non-typhoidal fevers. The seroincidence of *S* Typhi in Blantyre suggests that up to 43 times more people have asymptomatic or subclinical infections than those with blood-culture-detected typhoid fever.^14^ Furthermore, it is estimated that around 0·15–0·7% of the population in endemic regions are chronic carriers of *S* Typhi.^40^ These rates represent a high burden of asymptomatic, subclinical, or chronic infections, which could be evolving in response to ciprofloxacin given because of fever induced by a different cause. Environmental factors, such as water sources, represent a second niche for ciprofloxacin exposure. However, we suspect that there would be a longer delay between ciprofloxacin use and resistance emergence from this indirect exposure than our study indicated. A precise point-of-care diagnostic for typhoid fever could lower ciprofloxacin use in Malawi by ensuring that it is used only for *S* Typhi-related fevers. We urge the development and introduction of enhanced diagnostics in high-incidence areas to refine antibiotic use, potentially mitigating the emergence of resistance.

Ultimately, reducing the burden of disease through systemic measures such as the introduction of efficacious vaccines (eg, typhoid conjugate vaccines [TCVs]) into high-burden settings and water, sanitation, and hygiene (WASH) interventions are likely to be the most comprehensive solutions to preventing the emergence of DCS *S* Typhi.^39^ Indeed, TCVs were introduced into Zimbabwe in response to an outbreak of typhoid there in 2018 that included a large proportion of ciprofloxacin non-susceptible isolates.^12^^,^^13^ Of note, Malawi also conducted a national campaign and introduced TCVs in May, 2023, for all children aged 9 months to 15 years, and it is hoped that ongoing surveillance will show whether these measures help to reduce the future burden of AMR among *S* Typhi.

In conclusion, we have shown an association between rising empirical ciprofloxacin prescriptions and the proportion of *S* Typhi isolates with QRDR mutations in the following month. If this association is causal, which we believe is probable, then it highlights the speed with which bacterial populations can respond to changes in drug pressure. In our setting, where only around 1% of febrile patients have culture-positive typhoid fever, improved diagnostics would help to reduce ciprofloxacin prescriptions and prevent resistance from emerging, while effective vaccination and improved WASH interventions could also reduce the burden of disease and this driver of empirical antibiotic prescribing.

## Data sharing

Phylogenetic trees and annotation files are available from FigShare. Code to reproduce the statistical analysis and generate the plots can be accessed at GitHub. Genome accessions and relevant patient data are available in appendix 2.

## Declaration of interests

We declare no competing interests.
