# Supplementary material for: Evaluating the relationship between ciprofloxacin prescription and non-susceptibility in Salmonella Typhi in Blantyre, Malawi: an observational study
Source: Lancet Microbe. 2024 Mar;5(3):e226–34. doi: 10.1016/S2666-5247(23)00327-0 (PMC10914669; doi:10.1016/S2666-5247(23)00327-0)
Supplement: Supplementary appendix 1 [file mmc1.pdf]

THE LANCET
Microbe

Supplementary appendix 1

This appendix formed part of the original submission and has been peer reviewed.
We post it as supplied by the authors.

Supplement to: Ashton PM, Chunga Chirambo A, Meiring JE, et al. Evaluating the relationship between ciprofloxacin prescription and non-susceptibility in *Salmonella* Typhi in Blantyre, Malawi: an observational study. *Lancet Microbe* 2024. https://doi.org/10.1016/S2666-5247(23)00327-0

Supplementary Methods

PubMed query for Research in Context section

(Afghanistan OR Angola OR Benin OR Botswana OR Burkina Faso OR Burundi OR Cambodia OR Cameroon OR Central African Republic OR Chad OR Comoros OR Congo OR Djibouti OR Equatorial Guinea OR Eritrea OR Eswatini OR Ethiopia OR Gabon OR Gambia OR Ghana OR Guinea OR Guinea-Bissau OR Haiti OR India OR Kenya OR Kiribati OR Lesotho OR Liberia OR Madagascar OR Malawi OR Mali OR Mauritania OR Mozambique OR Myanmar OR Namibia OR Niger OR Nigeria OR Pakistan OR Papua New Guinea OR Philippines OR Rwanda OR Senegal OR Sierra Leone OR Solomon Islands OR Somalia OR South Africa OR South Sudan OR Sudan OR Togo OR Uganda OR Vanuatu OR Yemen OR Zambia OR Zimbabwe OR Cote d'Ivoire OR Democratic Republic of the Congo OR Lao OR Micronesia OR Timor OR Tanzania) AND (fluoroquinolones[Title/Abstract] OR ciprofloxacin[Title/Abstract] or quinolone[Title/Abstract]) AND (E. coli [Title/Abstract] OR Salmonella [Title/Abstract] OR Enterobacteriaceae [Title/Abstract] OR Klebsiella [Title/Abstract] OR enteric [Title/Abstract] OR bacteria* [Title/Abstract]]) NOT (tuberculosis[Title/Abstract] OR TB[Title/Abstract]) AND (usage [Title/Abstract] OR risk factors [Title/Abstract] or prescriptions [Title/Abstract]) AND (resistance [Title/Abstract] OR susceptibility [Title/Abstract]) NOT (review[Publication Type]))

Setting

Ndirande township is an informal urban settlement with a population of around 100,000 people. Population density is around 15,000 people per km^2, and approximately 85% of residents drink water from shallow wells, rain water, or boreholes, with small numbers having access to municipal water supplies (Kamanula, Zambasa, and Masamba 2014). Zingwanga is a similar setting to Ndirande. QECH, the largest government hospital in Malawi, has 1350 beds

and provides free secondary and tertiary healthcare to the approximately 1.3 million residents of Blantyre District and acts as a tertiary referral centre for the Southern Region of Malawi. It admits approximately 10,000 adult and 25,000 paediatric patients per year.

Sampling

Individuals who were resident in the demarcated geographical area for the STRATAA study (i.e. Ndriande township) or had been enrolled into the TyVAC trial and received a study vaccine were encouraged to present to one of the study facilities when they developed a fever. The STRATAA study enrolled participants from a demographic census of 102,242 individuals in Ndirande township (Meiring et al. 2021) whilst the TyVAC trial enrolled and vaccinated 28,130 children from within the Ndirande and Zingwangwa townships (Patel et al. 2021). A schematic diagram outlining the relationship between the STRATAA and TyVAC cohorts can be seen in Supplementary Figure 1. Participants who received either the candidate or placebo vaccine from the TyVAC trial, or participants from the censused areas (STRATAA study) who presented to a study passive surveillance site and reported a history of fever for ≥72 hours or had a measured axillary temperature of ≥38.0ºC, were recruited by members of the passive surveillance study team.

Participants had clinical data collected along with 3-5 ml of blood for microbiological culture. Microbiological culture of blood from all sites/studies was performed at the MLW laboratory on all collected samples with an automated system (BD BACTEC Blood Culture System [Becton-

Dickinson, Franklin Lakes, NJ, USA] or BacT/ALERT [BioMerieux, Marcy-l'Étoile, France]) after collection of a single aerobic bottle. This study includes participants from STRATAA or TyVAC recruited between 01/10/2016 and 31/10/2019. Participants were provided with standard of care clinical management at the health centres, with the additional benefit of receiving a diagnostic blood culture. If required, participants were referred to QECH for inpatient care. If participants were blood-culture positive, they were contacted by the clinical team to ensure they remained well and were on the correct antimicrobial therapy. All enrolled participants had antimicrobial usage for the two weeks prior to enrollment and antimicrobial prescriptions on the day of enrollment recorded in the electronic case record form.

Microbiology

Isolates were cultured on Mueller-Hinton agar with a 5 µg pefloxacin disk (Fisher Scientific, UK), any isolate with a zone of inhibition less than 24mm was considered to be resistant and the ciprofloxacin phenotype determined by E-test. Ciprofloxacin E-tests were performed on Mueller-Hinton agar according to the manufacturer's instructions (BioMerieux, Marcy-l'Étoile, France), isolates with an MIC of >0.06-1 µg were considered to have decreased ciprofloxacin susceptibility, isolates with an MIC >1 µg were considered to be resistant (Hassing et al. 2013).

Bioinformatics

Sequencing data were trimmed using bbduk v38.96 (‘BBMap’ n.d.) in order to remove adapters and low-quality sequencing regions `bbduk.sh ref=adapters.fa in=R1.fastq in2=R2.fastq out=R1.trimmed.fastq.gz out2=R2.trimmed.fastq.gz ktrim=r k=23 mink=11 hdist=1 tbo tpe

qtrim=r trimq=20 minlength=50`. Trimmed FASTQs were analysed with mykrobe v0.10.0 using the docker image `flashton/mykrobe_for_typhi:latest` and the GenoTyphi panel i.e. `--species typhi` (Bradley et al. 2015; Dyson and Holt 2021) to identify the sub-clade of *S.* Typhi. Antimicrobial resistance genes and mutations were identified using amr-finder-plus v3.9.8 with the `-O Salmonella` option (Feldgarden et al. 2019).

Trimmed FASTQs were also mapped against the CT18 reference genome (NCBI accession AL513382.1) using bwa mem v0.7.17-r1198-dirty (Li 2013). SNPs were called with GATK version 3.8-1-0-gf15c1c3ef in unified genotyper mode (Van der Auwera and O'Connor 2020). Positions where the majority allele accounted for < 90% of reads mapped at that position, which had a genotype quality of <30, depth <5x, or mapping quality <30 were recorded as Ns in further analyses. A consensus genome was generated for each genome. These steps were carried out using the PHEnix pipeline https://github.com/phe-bioinformatics/PHEnix. Maximum likelihood phylogenies were then created using IQ-TREE v1.6.12 with in-built model selection (Minh et al. 2020; Kalyaanamoorthy et al. 2017). Phylogenetic trees were annotated using iTOL v6.8 (Letunic and Bork 2019).

Statistical analysis

Analysis was done in R version 4.1.0 and links to a Github repository containing R code to reproduce all analyses is available in the Data Availability section of the manuscript.

Equation 1:

$$g(E[Y|X]) = \beta_0 + \beta_1 X$$

where Y is a binomial random variable recording the QRDR typhoid status for a given sample, X is recording the total number of ciprofloxacin prescriptions in the preceding month and g is the logit function, i.e. $g(x) = log\left(\frac{x}{1-x}\right)$. The data were complete with respect to the variables included in the analysis (proportion of QRDR S. Typhi and monthly ciprofloxacin prescriptions was available for each month) and hence no specific missing data methods were needed. The binomial 95% confidence intervals for percentage proportions were calculated using the prop.test function in R. We have interpreted odds ratios as relative risk given the rarity of the outcome (S. Typhi with QRDR).

Supplementary references

'BBMap'. n.d. SourceForge. Accessed 23 July 2022. https://sourceforge.net/projects/bbmap/.

Bradley, Phelim, N. Claire Gordon, Timothy M. Walker, Laura Dunn, Simon Heys, Bill Huang, Sarah Earle, et al. 2015. 'Rapid Antibiotic-Resistance Predictions from Genome Sequence Data for Staphylococcus Aureus and Mycobacterium Tuberculosis'. *Nature Communications* 6 (1). https://doi.org/10.1038/ncomms10063.

Dyson, Zoe A., and Kathryn E. Holt. 2021. 'Five Years of GenoTyphi: Updates to the Global Salmonella Typhi Genotyping Framework'. *The Journal of Infectious Diseases* 224 (12 Suppl 2): S775–80. https://doi.org/10.1093/infdis/jiab414.

Feldgarden, Michael, Vyacheslav Brover, Daniel H. Haft, Arjun B. Prasad, Douglas J. Slotta, Igor Tolstoy, Gregory H. Tyson, et al. 2019. 'Validating the AMRFinder Tool and Resistance Gene Database by Using Antimicrobial Resistance Genotype-Phenotype Correlations in a Collection of Isolates'. *Antimicrobial Agents and Chemotherapy* 63 (11). https://doi.org/10.1128/AAC.00483-19.

Hassing, R.-J., W. H. F. Goessens, D. J. Mevius, W. Pelt, J. W. Mouton, A. Verbon, and P. J. Genderen. 2013. 'Decreased Ciprofloxacin Susceptibility in Salmonella Typhi and Paratyphi Infections in Ill-Returned Travellers: The Impact on Clinical Outcome and Future Treatment Options'. *European Journal of Clinical Microbiology & Infectious Diseases* 32 (10): 1295–1301. https://doi.org/10.1007/s10096-013-1878-9.

Kalyaanamoorthy, Subha, Bui Quang Minh, Thomas K. F. Wong, Arndt von Haeseler, and Lars S. Jermiin. 2017. 'ModelFinder: Fast Model Selection for Accurate Phylogenetic Estimates'. *Nature Methods* 14 (6): 587–89. https://doi.org/10.1038/nmeth.4285.

Kamanula, John F., Omega J. Zambasa, and Wellington R.L. Masamba. 2014. 'Quality of Drinking Water and Cholera Prevalence in Ndirande Township, City of Blantyre, Malawi: Transboundary Water Cooperation: Building Partnerships.' *Physics and Chemistry of the Earth* 72–75: 61–67. https://doi.org/10.1016/j.pce.2014.09.001.

Letunic, Ivica, and Peer Bork. 2019. 'Interactive Tree Of Life (ITOL) v4: Recent Updates and

New Developments'. *Nucleic Acids Research* 47 (W1): W256–59. https://doi.org/10.1093/nar/gkz239.
Li, Heng. 2013. 'Aligning Sequence Reads, Clone Sequences and Assembly Contigs with BWA-MEM'. *ArXiv:1303.3997 [q-Bio]*, March. http://arxiv.org/abs/1303.3997.
Meiring, James E., Mila Shakya, Farhana Khanam, Merryn Voysey, Maile T. Phillips, Susan Tonks, Deus Thindwa, et al. 2021. 'Burden of Enteric Fever at Three Urban Sites in Africa and Asia: A Multicentre Population-Based Study'. *The Lancet Global Health* 9 (12): e1688–96. https://doi.org/10.1016/S2214-109X(21)00370-3.
Minh, Bui Quang, Heiko A Schmidt, Olga Chernomor, Dominik Schrempf, Michael D Woodhams, Arndt von Haeseler, and Robert Lanfear. 2020. 'IQ-TREE 2: New Models and Efficient Methods for Phylogenetic Inference in the Genomic Era'. *Molecular Biology and Evolution* 37 (5): 1530–34. https://doi.org/10.1093/molbev/msaa015.
Patel, Priyanka D., Pratiksha Patel, Yuanyuan Liang, James E. Meiring, Theresa Misiri, Felistas Mwakiseghile, J. Kathleen Tracy, et al. 2021. 'Safety and Efficacy of a Typhoid Conjugate Vaccine in Malawian Children'. *New England Journal of Medicine* 385 (12): 1104–15. https://doi.org/10.1056/NEJMoa2035916.
Van der Auwera, Geraldine A., and Brian D. O'Connor. 2020. *Genomics in the Cloud: Using Docker, GATK, and WDL in Terra*. First edition. Sebastopol, CA: O'Reilly Media.

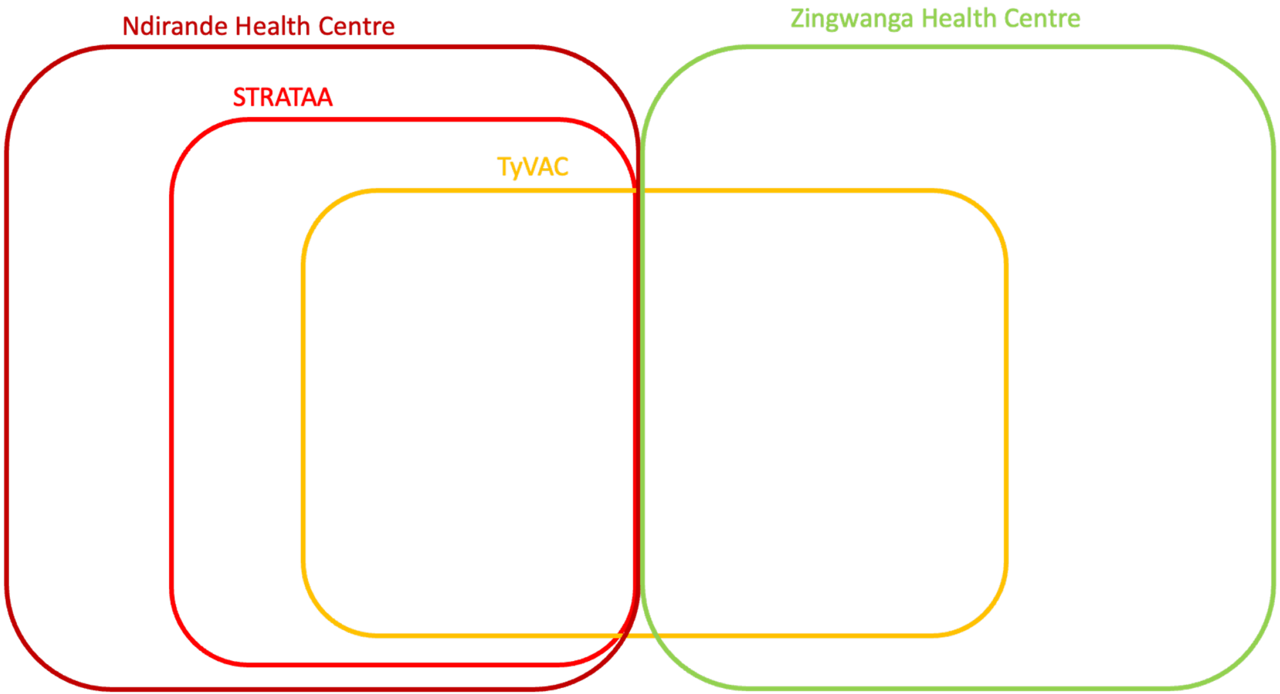

Supplementary Figure 1: A schematic diagram demonstrating the relationship between the STRATAA and TyVAC cohorts, and their recruitment from Ndirande and Zingwangwa. TyVAC recruited from both Ndirande and Zingwanga, while STRATAA only recruited from Ndirande. All TyVAC participants from Ndirande were also enrolled in STRATAA. TyVAC participants from Ndirande were only those enrolled in the typhoid conjugate vaccine study, while all residents were eligible for enrollment in STRATAA (assuming they met the fever criteria etc).

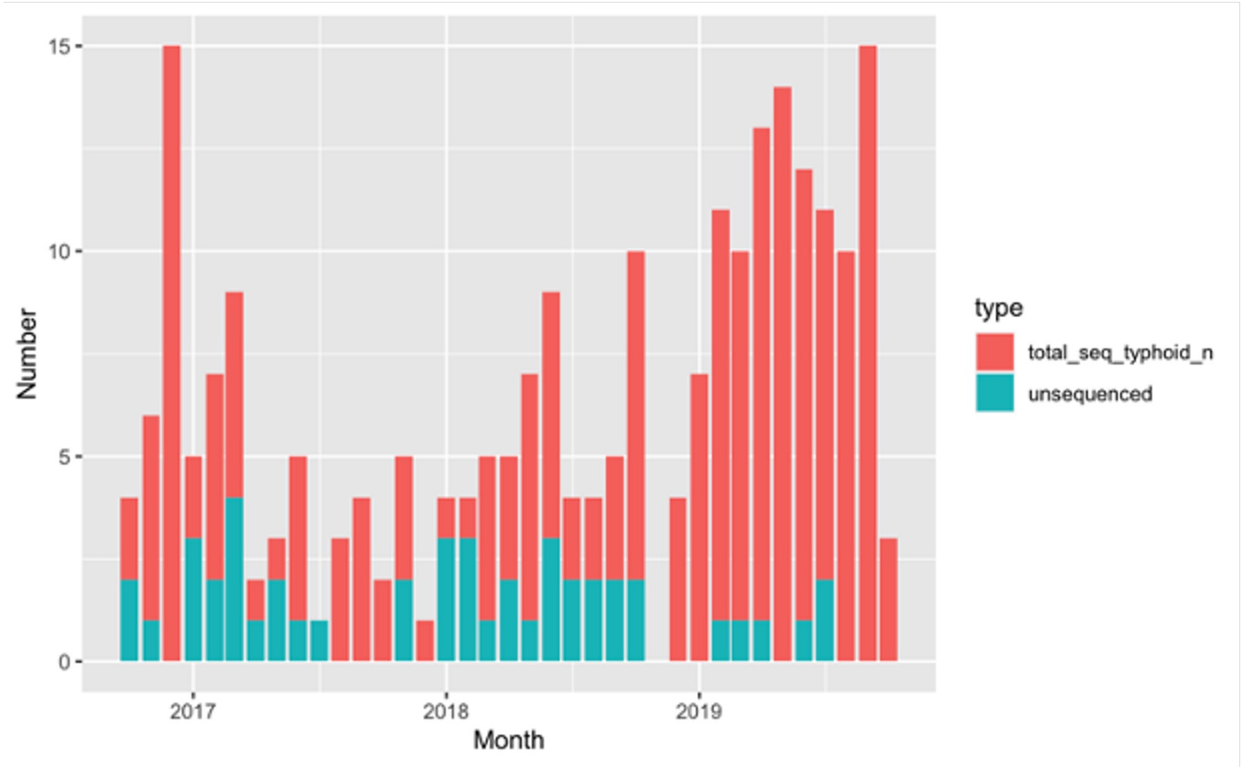

Supplementary Figure 2: The number of patients with sequenced and unsequenced *S.* Typhi isolates across the recruitment period

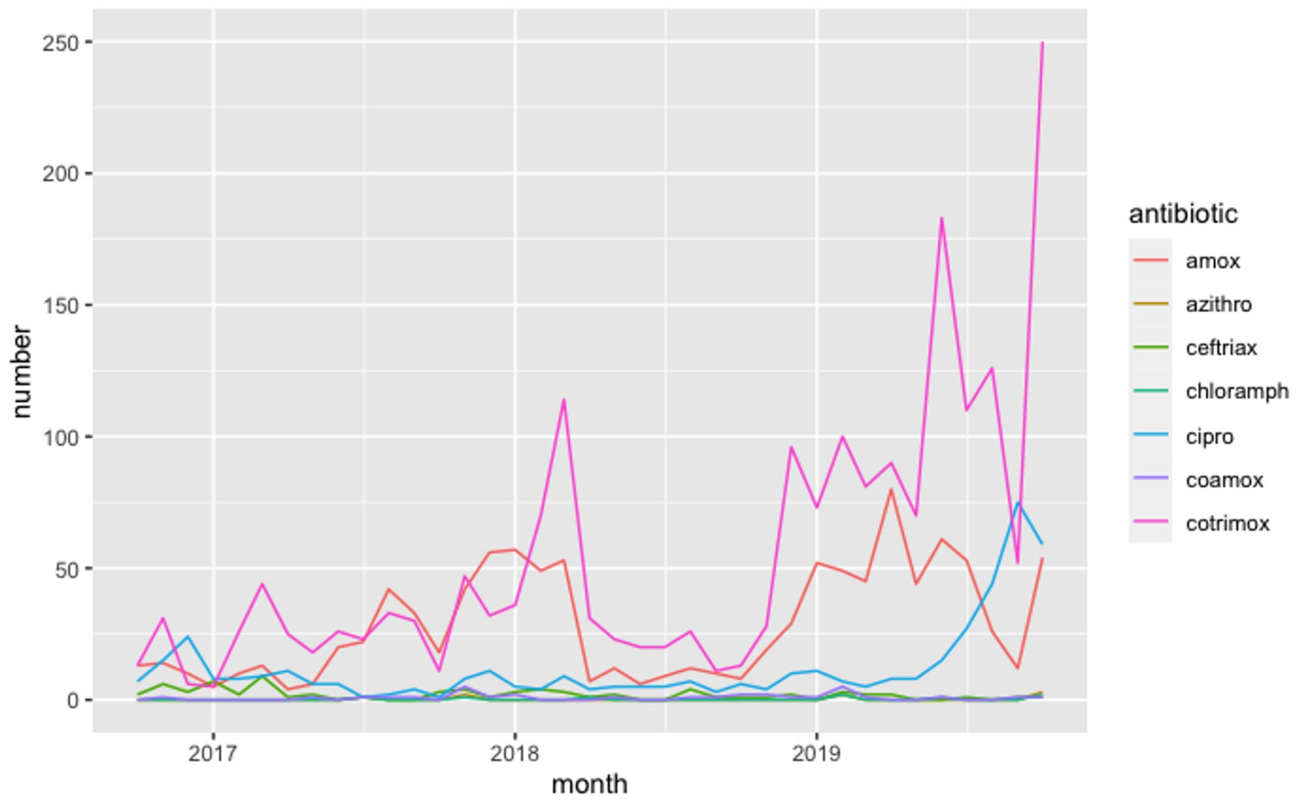

Supplementary Figure 3: Monthly prescriptions of 7 antibiotics to STRATAA participants

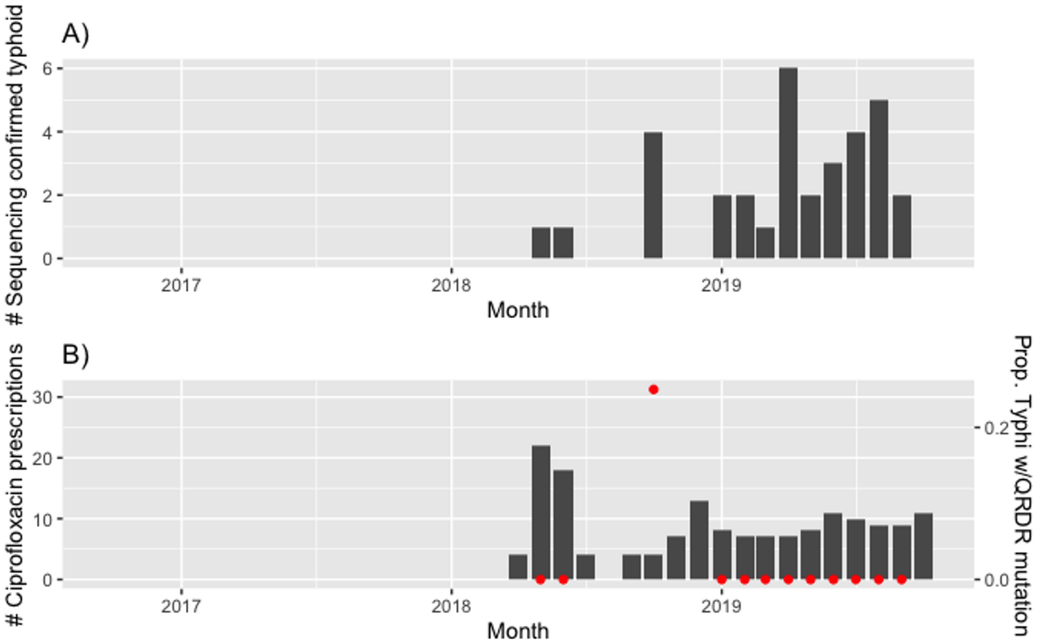

Supplementary Figure 4: Zingwangwa Health Centre data A) Total sequencing confirmed typhoid cases B) the number of ciprofloxacin prescriptions (grey bars) and the proportion of sequencing confirmed S. Typhi with QRDR mutations (red dots)

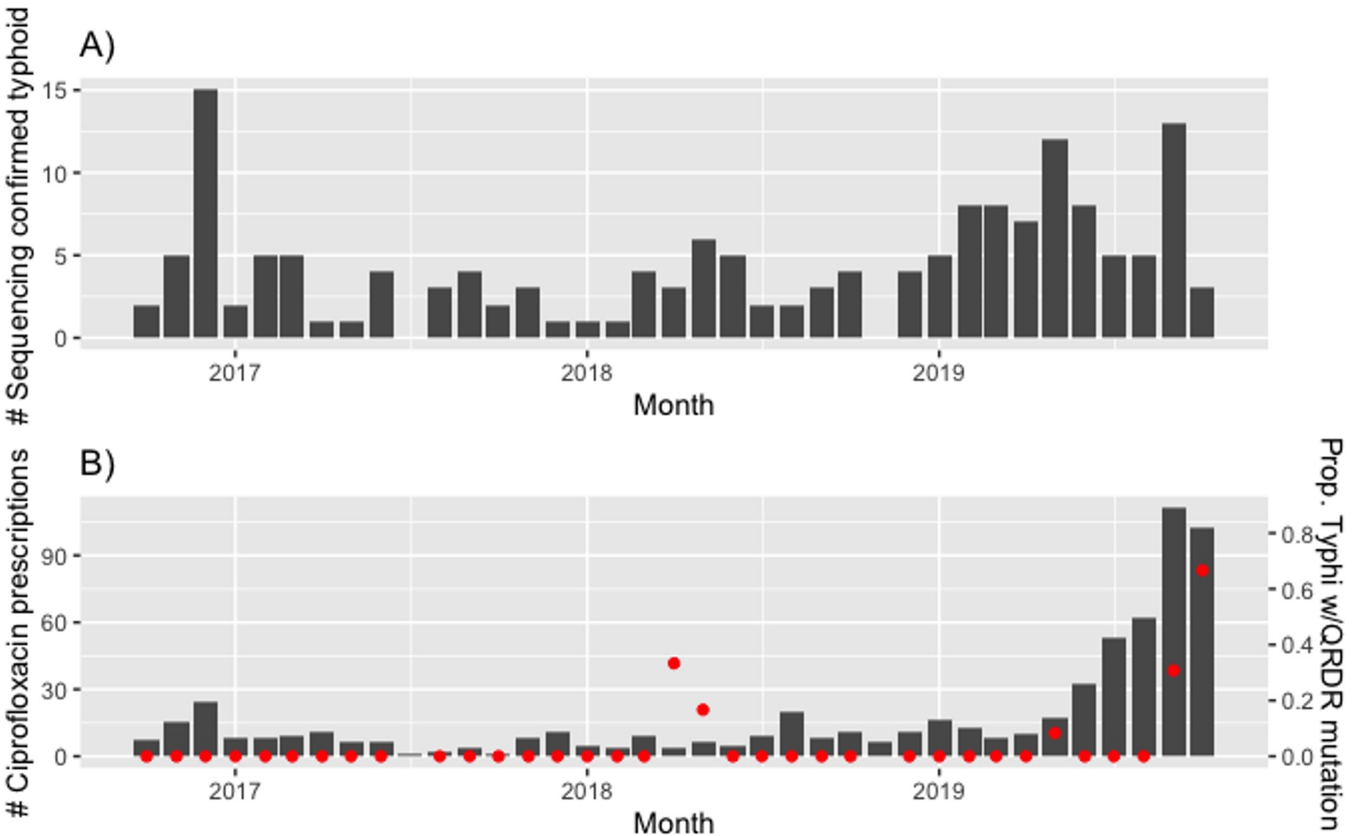

Supplementary Figure 5: Ndirande Health Centre data A) Total sequencing confirmed typhoid cases B) the number of ciprofloxacin prescriptions (grey bars) and the proportion of sequencing confirmed S. Typhi with QRDR mutations (red dots)

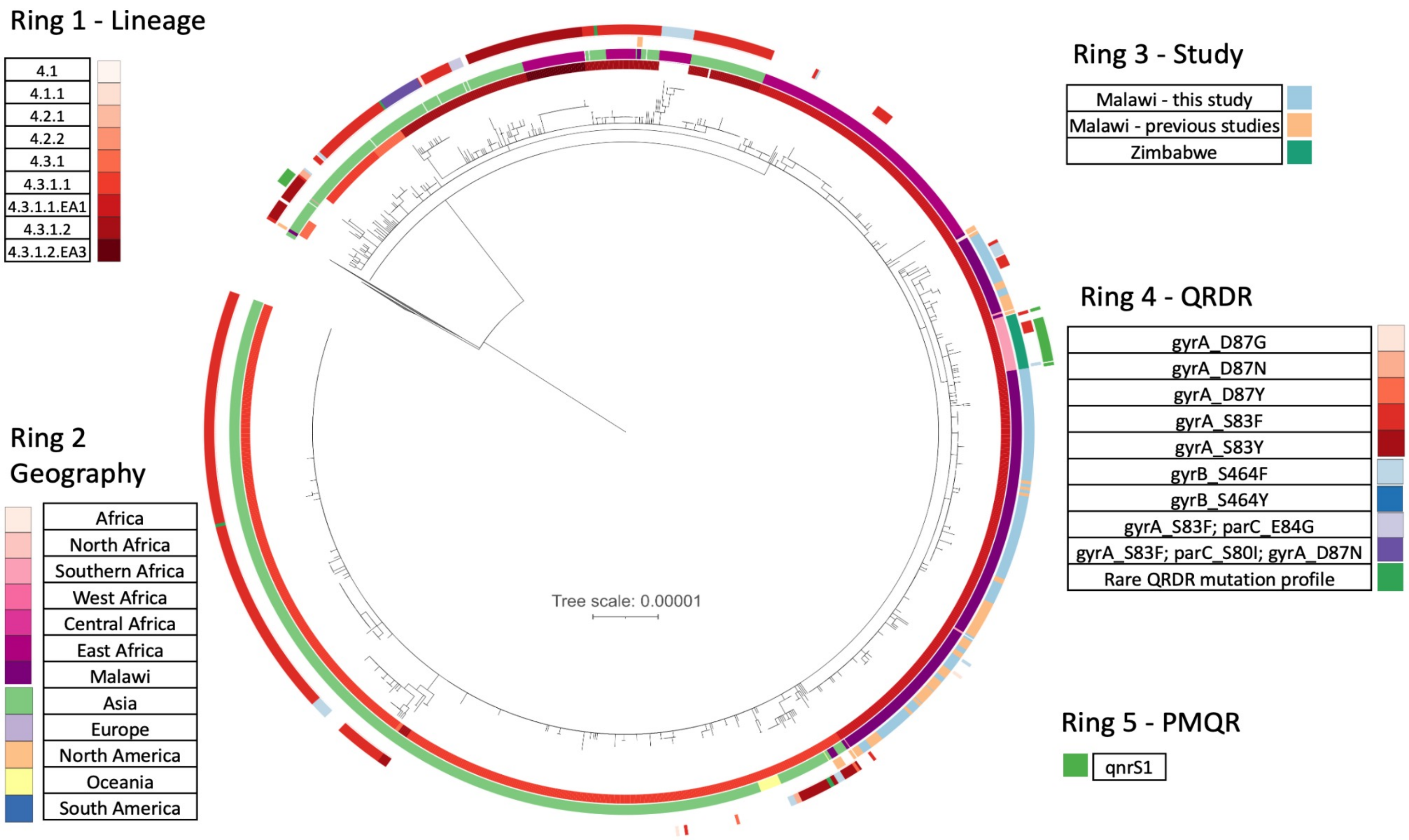

Supplementary Figure 6: Phylogenetic tree of 1176 H58 S. Typhi genomes, placing those from Blantyre in the global context of other H58 samples
